# N-Methyl D-Aspartate Receptor Antagonist Kynurenic Acid Affects Human Cortical Development

**DOI:** 10.3389/fnins.2016.00435

**Published:** 2016-09-30

**Authors:** Inseyah Bagasrawala, Nada Zecevic, Nevena V. Radonjić

**Affiliations:** ^1^Department of Neuroscience, University of Connecticut HealthFarmington, CT, USA; ^2^Department of Psychiatry, University of Connecticut HealthFarmington, CT, USA

**Keywords:** human fetal brain, cerebral cortex, tissue culture, KYNA, NMDARs, neurogenesis, cortical progenitor cells

## Abstract

Kynurenic acid (KYNA), a neuroactive metabolite of tryptophan degradation, acts as an endogenous N-methyl-D-aspartate receptor (NMDAR) antagonist. Elevated levels of KYNA have been observed in pregnant women after viral infections and are considered to play a role in neurodevelopmental disorders. However, the consequences of KYNA-induced NMDAR blockade in human cortical development still remain elusive. To study the potential impact of KYNA on human neurodevelopment, we used an *in vitro* system of multipotent cortical progenitors, i.e., radial glia cells (RGCs), enriched from human cerebral cortex at mid-gestation (16–19 gestational weeks). KYNA treatment significantly decreased RGCs proliferation and survival by antagonizing NMDAR. This alteration resulted in a reduced number of cortical progenitors and neurons while number and activation of astrocytes increased. KYNA treatment reduced differentiation of RGCs into GABAergic neurons, while differentiation into glutamatergic neurons was relatively spared. Furthermore, in mixed cortical cultures KYNA triggered an inflammatory response as evidenced by increased levels of the pro-inflammatory cytokine IL-6. In conclusion, elevated levels of KYNA play a significant role in human RGC fate determination by antagonizing NMDARs and by activating an inflammatory response. The altered cell composition observed in cell culture following exposure to elevated KYNA levels suggests a mechanism for impairment of cortical circuitry formation in the fetal brain after viral infection, as seen in neurodevelopmental disorders such as schizophrenia.

## Introduction

Kynurenic acid (KYNA) is an intermediate metabolite of the kynurenine pathway and the only naturally occurring antagonist of the glutamatergic NMDA receptor (NMDAR) in the human brain (Stone, 2013). In the brain, KYNA is synthesized in astrocytes by the irreversible transamination of L-kynurenine, the first major catabolic product of tryptophan. Elevated levels of KYNA have been found in the cerebrospinal fluid and in post-mortem brains of adult schizophrenia (Sch) patients (Erhardt et al., 2001; Schwarcz et al., 2001; Sathyasaikumar et al., 2011; Holtze et al., 2012). Both stress and infections, in rats activates indoleamine 2,3 dioxygenase (IDO), a cytokine responsive enzyme that catalyzes the formation of kynurenine, which may impair brain development (Pocivavsek et al., 2014; Notarangelo and Pocivavsek, 2016). Furthermore, environmental insults during development, such as maternal influenza infection, increase the risk for Sch and related disorders (Wright et al., 1995; Stöber et al., 2002; Limosin et al., 2003; Brown, 2012). In rodents, KYNA can cross the placental and fetal blood-brain barriers (Heyes et al., 1990; Scharfman and Goodman, 1998) and induce secretion of various pro-inflammatory cytokines, such as interferon gamma (IFN-γ) and interleukin 6 (IL-6), from fetal astrocytes (Meyer et al., 2011). Additionally, *in vitro* studies have demonstrated that IFN-γ and IL-6 can activate human fetal astrocytes to synthesize increased levels of KYNA (Guillemin et al., 2001). Thus, exposure of the fetal brain to KYNA may establish a positive feed-back loop, whereby KYNA levels are further enhanced (Guillemin et al., 2001; Meyer et al., 2011; Schwieler et al., 2015).

Glutamate acting via NMDARs has a trophic effect during development, and may play an important role in determining the selective survival of neurons and their proper connections (LoTurco et al., 1991; Haydar et al., 2000; Balasz, 2006). This is particularly pertinent to a possible role for disturbed NMDAR function in Sch, as an alteration or reduction of NMDARs has been demonstrated in medication-free Sch patients (Akbarian et al., 1996; Pilowsky et al., 2006), and abnormal glutamatergic activity has been reported in the pathophysiology of Sch (Deutsch et al., 1989; Coyle, 1996; Belforte et al., 2010). Furthermore, NMDARs are abundantly expressed in the developing human cerebral cortex at midgestation (Bagasrawala et al., 2016) Many animal models of Sch mimic a transient NMDAR hypofunction during development using NMDAR antagonists such as MK-801 (Ikonomidou et al., 1999), phencyclidine (PCP; Wang et al., 2001; Radonjić et al., 2008) and ketamine (Breier et al., 1997; Rujescu et al., 2006).

Even though it is known that there is a higher incidence of Sch in people exposed to viral infections *in utero* (Bale et al., 2010; Brown and Derkits, 2010; Selemon and Zecevic, 2015), presumably due to an inflammatory response and increased levels of KYNA, it is still unclear how elevated levels of KYNA affect the developing human brain. The goal of this study was to elucidate the role of KYNA as an endogenous NMDAR antagonist in the human fetal brain. As experimental manipulation of the developing human brain *in vivo* is not possible, we established an *in vitro* system to test the effects of KYNA on human cortical progenitor cells.

Radial glia cells (RGCs) are multipotent cortical progenitor cells capable of generating all neural cell types, including subpopulations of intermediate and interneuron progenitors, neurons, astrocytes and oligodendrocytes (Howard et al., 2006; Mo et al., 2007; Mo and Zecevic, 2009; Hansen et al., 2010, 2013; Lui et al., 2011; Yu and Zecevic, 2011; Ma et al., 2013; Radonjić et al., 2014). Specifically, we were interested in how exposure to KYNA affects survival, proliferation, and specification of RGCs into various cell types, including cortical interneurons and pyramidal cells. We demonstrated that elevated levels of KYNA, through a NMDAR blockade, not only reduced survival and proliferation of RGCs, but significantly altered the progeny of cortical RGCs. Treatment with KYNA promoted gliogenesis at the expense of neurogenesis, and increased activation of astrocytes. Moreover, we detected increased levels of a pro-inflammatory cytokine, IL-6, which suggests an initiation of an inflammatory response. The combined effects of a NMDAR blockade and an inflammatory response could impair cortical circuitry formation *in utero* and in so doing contribute to the pathophysiology of neurodevelopmental illness, possibly including the adult-onset disorder of schizophrenia.

## Materials and methods

### Human fetal brain tissue

Human fetal brain tissue (*n* = 8), free of any developmental abnormalities, at 16–19 gw was obtained with written informed consent and the approval of University of Connecticut Health Ethics Committee, from the Human Developmental Biology Resource (Newcastle University, Newcastle upon Tyne, England) and the Tissue Repository of The Albert Einstein College of Medicine (Bronx, NY, USA), (Table 1). Transport of the human material was done on ice, following all necessary requirements and regulations set by the Institutional Ethics Committees, of our institution and respective tissue repositories.

**Table 1:** **Description of human fetal tissue and methods used**.

**Case number**	**Sex**	**Gestational week**	**Method used**
1	MALE	16	qPCR, Western blot, Immunocytochemistry, ELISA
2	MALE	17	qPCR, Western blot, Immunocytochemistry, ELISA
3	MALE	18	qPCR, Western blot, Immunocytochemistry, ELISA
4	MALE	19	qPCR, Western blot, Immunocytochemistry, ELISA
5	MALE	16	Immunocytochemistry (Mixed cells), ELISA
6	MALE	18	Immunocytochemistry (Mixed cells), ELISA
7	MALE	18	Immunocytochemistry (Mixed cells), ELISA
8	MALE	19	Immunocytochemistry (Mixed cells), ELISA

The age of the tissue was determined by the following criteria: crown-rump length, weeks after ovulation, and anatomical landmarks. The tissue was transported on ice in Hank's Balanced Salt Solution (HBSS; Life Technologies, Grand Island, NY, USA) from the aforementioned brain repositories to the lab. A small unfixed piece (1 cm^2^) of the dorsal telencephalic region was used to generate dissociated cell cultures (Zecevic et al., 2005; Radonjić et al., 2014). We always tried to used multiple methods on the same case, but not all the cases were available for every method used in this study, as specified in Table 1.

### Dissociated mixed cell culture and enrichment of RGCs

**Figure 1:**
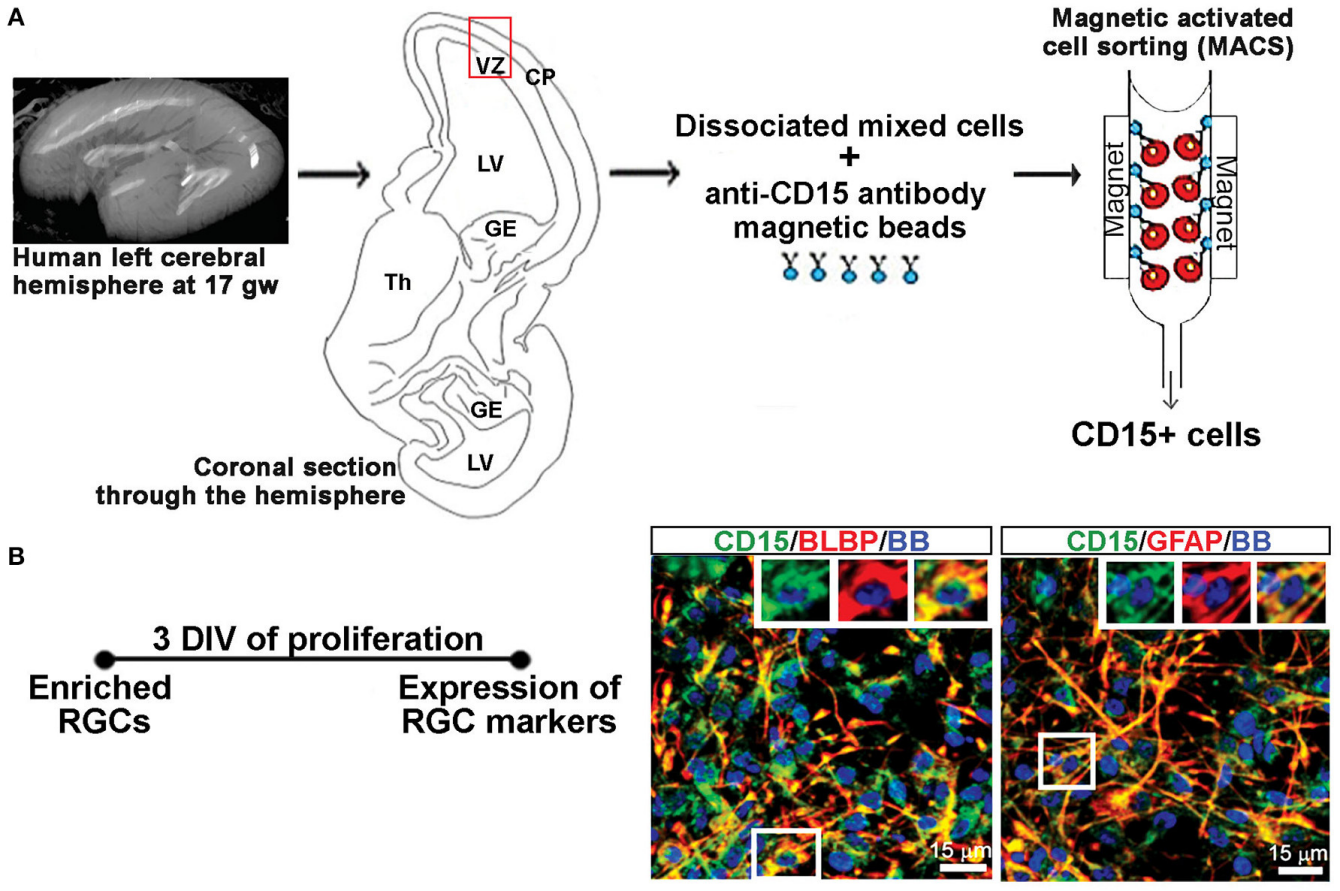
**Enriched RGCs from human fetal cortex using MACS. (A)** Dissociated mixed cell cultures are established from the telencephalic wall (red box), and RGCs are enriched from these cultures through magnetic sorting (MACS) using anti-CD15 micro-beads. **(B)** Enriched RGCs are co-labeled for CD15 and radial glia markers BLBP or GFAP after 3 DIV of proliferation. Nuclear stain: bisbensimide (BB).

A previously published protocol (Mo and Zecevic, 2009) was used to establish dissociated mixed cell cultures. As briefly described here, the isolated tissue was dissociated mechanically and enzymatically at 37°C for 30 min with 0.025% trypsin (Gibco, Beverly, MA, USA), followed by addition of DNase (Sigma-Aldrich, St Louis, MO, USA; 2 mg/mL), washing with HBSS (Life Technologies) and suspension in proliferation medium (PM; Figure 1A). The PM consisted of DMEM/F12 (Life Technologies) supplemented with 10 ng/mL of basic fibroblast growth factor (bFGF, Peprotech, Rocky Hill, NJ, USA), 10 ng/mL of epidermal growth factor (EGF, Millipore, Billerica, MA, USA), and B27 (Life Technologies). Seven to ten days after plating, the cells proliferated in PM and reached 80% confluency whereupon they were used for experiments on activated astrocytes including sandwich ELISA, and to generate RGC cultures.

RGCs were isolated from mixed cell cultures using the cell surface glycan marker CD15 via magnetic columns (MACS, Miltenyi Biotec, Auburn, CA, USA) (Mo et al., 2007; Radonjić et al., 2014). Enriched RGCs were fixed after 2 hrs and co-labeled for surface antigen CD15 and either brain lipid binding protein (BLBP; 97.8%) or glial fibrillary acidic protein (GFAP; 99.1%), both markers of RGCs (Figure 1B), demonstrating the purity of enriched RGC cultures. The expression of the NR1 gene and protein in cell lysates as well as NR1 expression on different cell types using double immunostaining, was done after 3 days *in vitro* (DIV) in PM, and after 7 DIV in differentiation medium (DM), which is devoid of growth factors. RGCs plated on poly-D-lysine (Sigma-Aldrich) coated coverslips (12 mm) at a density of 250,000 cells/ml were used for immunocytochemistry. Poly-D-lysine coated six wells (10 cm^2^ growth area/well) plated with 2 million cells/ml were used to collect cell lysates for total protein and RNA isolation.

### Pharmacological treatments of cell cultures

RGCs cultures were treated with each of these substances: KYNA, NMDAR-specific antagonist D-amino phosphonovalerate (D-APV), and nicotinic acetylcholine receptor (nAChR) antagonist Tubocurarine chloride (TBC), to determine if the effect of KYNA was mediated through NMDARs or nAChRs. Concentration-response curves were first plotted 48 h after RGC cultures underwent treatments with eight different concentrations (0.001–100.0 μM) of KYNA (Sigma Aldrich), D-APV (Tocris Biosciences, Bristol, UK) and TBC. KYNA was dissolved in DMSO (Dimethyl sulphoxide, Sigma Aldrich), and therefore two sets of controls were maintained: (1) RGCs grown only in PM, (2) RGCs grown in PM containing DMSO. In all analyzed experiments there was no difference between control PM and PM+DMSO, hence PM+DMSO results were represented in graphs as the control. The effective concentration at which 50% of the cell population was affected (EC50) was determined in both assays to decide the treatment concentrations to be used for further experiments, where cells were analyzed after 3 DIV in PM, and after 7 DIV in DM.

### Cell survival assay

A live/dead assay (Molecular Probes, Eugene, OR, USA) was performed after 48 h of KYNA treatment to assess its effect on cell viability. The kit enables detection of live cells via the cell-permeant molecule calcein acetoxymethyl that gives a green fluorescence in the presence of intracellular esterases. Dead cells which have lost the intact cell membrane appear red due to incorporation of an ethidium homodimer into their degrading nucleic acids.

### Proliferation assay

At the end of the 48 h KYNA treatment period, cells were fixed and immunostained with anti-Ki67 antibody. Ki67 is a marker of cells undergoing mitotic divisions or proliferation. The percentage of Ki67^+^ cells was determined from the total number of cells marked with the nuclear stain bisbenzimide (BB).

### Immunocytochemistry (ICC)

Cells growing *in vitro* on coverslips were fixed with 4% paraformaldehyde, washed with PBS (phosphate buffer saline), blocked (0.2% bovine serum albumin (BSA), 0.01% Triton X-100, PBS) for 1 h at room temperature (RT), and incubated with primary antibodies (see Table 2) at 4°C overnight. The specificity of each primary antibody has been previously tested and shown in the data sheets obtained from the respective manufacturers. Specificity of staining was validated by replacing the primary antibody with the normal serum from the animal in which the antibody was produced, which resulted in the absence of fluorescent signal. The following day, fluorophore-conjugated secondary antibodies goat anti-mouse Alexa-488 (Jackson ImmunoResearch, Lot no. 104763), and goat anti-rabbit Alexa-555 (Molecular Probes, Catalog no. A21428) at a concentration of 1 μg/ml each were applied for 2 h at RT, followed by the nuclear stain BB for 1 min. All immunostained coverslips were mounted using the anti-fade reagent Fluoromount-G (Southern Biotech, Birmingham, AL, USA) to preserve the fluorescence.

**Table 2:** **List of primary antibodies used**.

**Primary antibody**	**Cell type identified**	**Species**	**Concentration**	**Catalog number**	**Company**
CD15	Radial Glia (RGC)	Mouse	2 μg/ml	#MS-1259-P	Thermo Scientific
BLBP	RGC	Rabbit	1.25 μg/ml	ab27171	Abcam
GFAP	RGC, Astrocyte	Mouse, rabbit	5 μg/ml	Z 0334	Dako
Tbr2	Intermediate Progenitor	Rabbit	2 μg/ml	ab23345	Abcam
Nkx2.1	Interneuron Progenitor	Rabbit	10 μg/ml	ab76013	Abcam
βIII tubulin	Neuron	Mouse, rabbit	2 μg/ml	065M4820V	Sigma-Aldrich
Tbr1	Glutamatergic Neuron	Rabbit	5 μg/ml	20932-1-AP	Proteintech
GABA	Interneuron	Rabbit	5 μg/ml	A2052	Sigma-Aldrich
CalR	Interneuron	Rabbit	1 μg/ml	7699/4	Swant
Ki67	Proliferating Cells	Rabbit	1 μg/ml	ab15580	Abcam
GAPDH	Housekeeping	Mouse	0.2 μg/ml	MAB374	Millipore
NR1	–	Mouse	10 μg/ml	75-272	NeuroMab

### Image analysis and statistical tests

Immunolabeled samples were visualized using an Axioscope microscope (Zeiss, Germany) equipped with Axiovision software and photographed using a digital camera with 63X lens. During acquisition of images of double immunolabeled sections the focus, brightness and contrast were constant. Twelve random images were taken from each coverslip and analyzed in Adobe Photoshop (v. 7.0), with consistent quality adjustments for contrast, brightness and color balance and used for cell counting.

We quantified cells labeled with each marker in its respective channel in Photoshop, as well as cells that show immunoreaction for both applied antibodies. The total cell number in the optical field was determined by quantifying cell nuclei labeled with a nuclear dye bisbensamide (BB). The percentage of double labeled cells was then calculated from a total cell count and averaged across the four coverslips/12 pictures/4 brains for each marker and each treatment and plotted in the bar graphs.

### Western blot (WB)

Cells were homogenized in lysis buffer (50 mM Tris–HCl pH 7.4, 150 mM NaCl, 1% NP-40, 1 mM phenylmethylsulfonyl fluoride, Sigma Aldrich, and protease inhibitor cocktail, Thermo Scientific, Agawam, MA, USA) on ice for 30 min, centrifuged at 14,000 × g for 15 min at 4°C, and the supernatants were collected as the cell lysates. The protein content of the samples was analyzed using the BCA protein estimation colorimetric assay (Thermo Scientific). Twenty micrograms of protein was loaded in each well and proteins were separated on 4–15% gradient polyacrylamide gels (Bio-Rad, Portland. ME, USA) at 110 v for 75 min. Samples were run, on three separate gels, to obtain results in triplicates. The separated proteins were transferred onto polyvinylidene fluoride (PVDF) membranes at 100 v for 60 min. A Ponceau stain on every blot, and a Coomassie stain for every gel was performed to confirm complete transfer of separated proteins. After blocking with 5% milk in TBS-T (1.0% Tween-20, PBS, 0.1% Triton X-100, distilled water; pH 7.4), the membrane (blot) was incubated with primary antibodies diluted in blocking solution (0.1 M Tris, 2% non-fat dry milk, 0.15 M NaCl, 0.01% Na-azide, pH 7.4) against the proteins of interest at 4°C (Table 2) overnight. The blot was washed three times, with 1X TBS-T (10% Tween, PBS, 0.1% TritonX-100, distilled water), and incubated with horseradish peroxidase (HRP)-conjugated secondary antibodies (Millipore) for 2 h at RT., Blots were incubated with SuperSignal West Dura Extended Duration Substrate (Thermo Scientific), and imaged on ChemiDoc MP (Bio-Rad) digital imaging system. The loading control for each blot was GAPDH (primary antibody dilution 1:5000). Each membrane was used only once, no stripping or re-probing has been performed. The density of each band was determined in Adobe Photoshop (v.7.0) using histogram analysis. The values obtained for each band were divided by the value of the corresponding GAPDH band. The averaged values obtained from KYNA treated groups were normalized to the control (DMSO) group to overcome individual differences between samples.

### Real-time polymerase chain reaction

Cells were treated with ice-cold TRIZOL® reagent (Invitrogen, Carlsbad, CA, USA; 1 ml/10 cm^2^ of surface area) and were broken down mechanically and through alternate freeze-thaw cycles. Chloroform (200 μl/1 ml of Trizol) was added to the homogenates, and tubes were vortexed, incubated (15 min at 4°C), and centrifuged (13,000 × g for 15 min at 4°C). The upper transparent phase was collected in sterile tubes, incubated with isopropanol (500 μl/tube; 20 min at RT), and centrifuged (13,000 × g for 15 min at 4°C). The pellets were incubated with 75% ethanol (4°C) and centrifuged (13,000 × g for 15 min). The pellets containing RNA were air dried and dissolved in 5% RNase Out solution. RNA concentrations were determined using the NanoDrop technology. cDNA was synthesized by reverse transcriptase-PCR using the First Strand Synthesis SuperScript III kit (Invitrogen). The SYBR Green (Applied Biosystems, Cheshire, UK) protocol was used to analyze the expression levels of mRNA from the cDNA samples (see Table 3 for list of genes and primers) by performing real time PCR using the CFX96 Connect® thermocycler (Bio-Rad). The real time PCR protocol involves a heating step to 95°C for 2 min followed by 40 cycles at 95°C for 15 s, at 55°C for 15 s, and at 68°C for 20 s. Cycle of threshold (*C*_*t*_) values were used to calculate the ΔΔ*C*_t_ values, and the formula 2^−ΔΔ*C*t^ was used to determine the mRNA expression level relative to the control sample which has an arbitrary value of 1.

**Table 3:** **List of primers used for qPCR**.

**Genes**	**Cell type identified**	**Forward primer sequence**	**Reverse primer sequence**
Nestin	RGC	5″-GCC CTG ACC ACT CCA GTT TA-3″	5″GGA GTC CTG GAT TTC CTT CC-3″
Tbr2	Intermediate progenitor	5″-GGG CAC CTA TCA GTA CAG CCA-3″	5″-AAG GAA ACA TGC GCC TGC-3″
Nkx2.1	Interneuron progenitor	5″-CAC GCA GGT CAA GAT CTG GTT-3″	5″-TTG CCG TCT TTC ACC AGG A-3″
Neurogenin	Neuron	5″-GCATCAAGA AGACACGCAGACTGA-3″	5″-TCTCGATCTTGGTTAGCTTGGCGT-3″
Tbr1	Glutamatergic neuron	5″-TCT GAG CTT CGT CAC AGT TTC-3″	5″-GCT GTT GTA GGC TCC GTT G-3″
Gad65/67	Interneuron	5″-CCT CAA CTA TGT CCG CAA GAC-3″	5″-TGT GCG AAC CCC ATA CTT CAA-3″
Gfap	Astrocyte	5″-CTG CGG CTC GAT CTG GTT-3″	5″-TCC AGC GAC TCA ATC TTC CTC-3″
Grin1	–	5″-CCA GTC AAG AAG GTG ATC TGC AC-3″	5″-TTC ATG GTC CGT GCC AGC TTG A-3″

### Sandwich enzyme linked immunosorbent assay (sandwich ELISA)

The eBioscience High Sensitivity human anti-IL6 ELISA (San Diego, CA) kit was used for the sandwich ELISA experiments on dissociated mixed cell cultures prepared from four fetal brains (16–19 gw). The assay was performed according to manufacturer's instructions. We used Origin to plot the standard curve and analyze results from the ELISA experiment.

### Statistics

All data were expressed as mean ± standard error of mean (SEM). Mean, SEM, and *p*-values were calculated in Microsoft Excel. For all experiments, data were averaged across the four brains (*n* = 4; 16–19 gw). Two-tailed Student's *t*-test was used to compare treated and control groups with statistical significance considered *p* ≤ 0.05.

## Results

### Cortical progenitors and neurons express NR1 at mid-gestation

**Figure 2:**
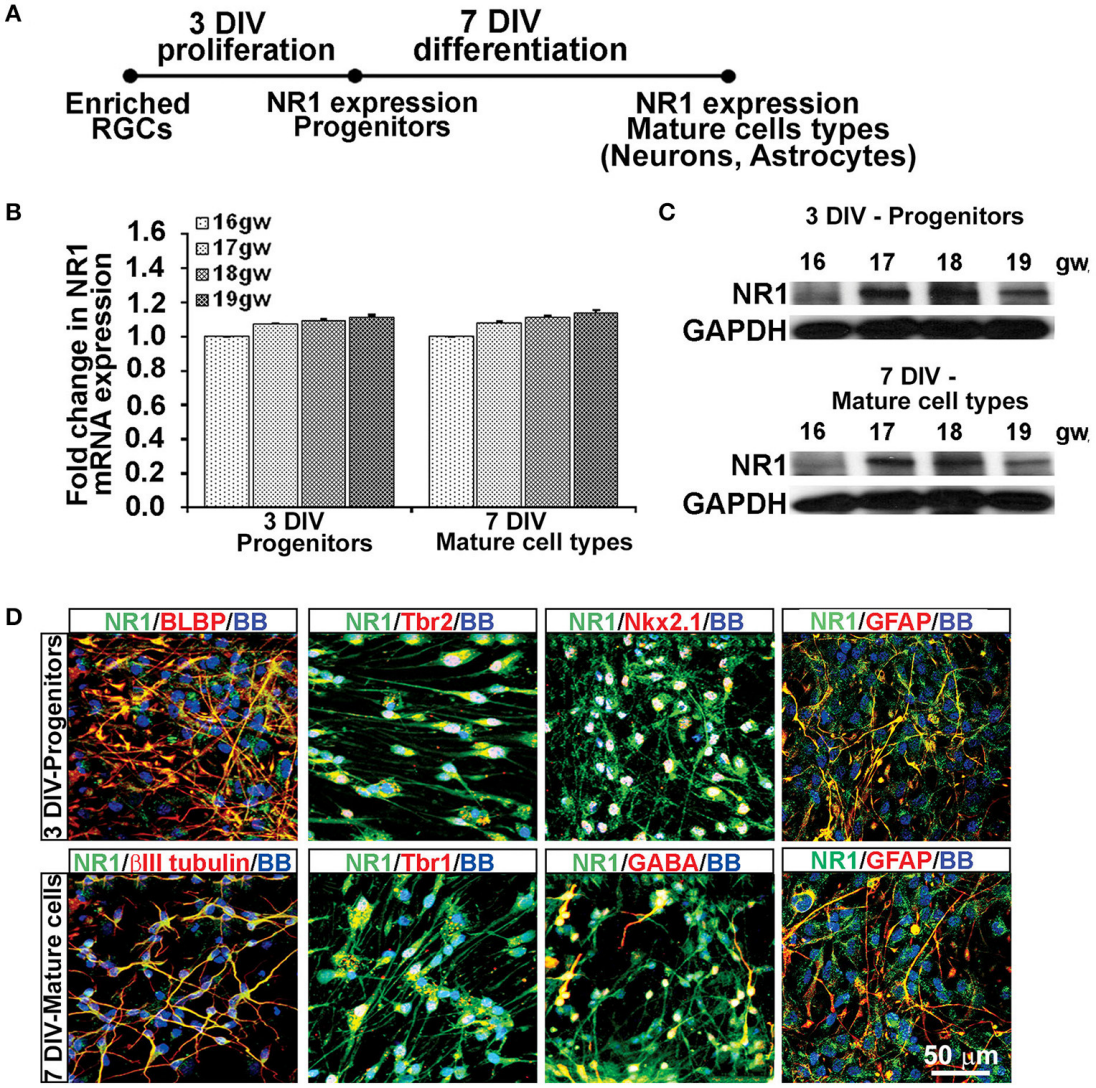
**Cell types that express NR1 subunit at mid-gestation. (A)** Timeline showing that NR1 expression was analyzed on progenitors after 3 DIV in PM and on mature cell types after 7 DIV in DM; *n* = 4. **(B)** qPCR and **(C)** Western blot showing NR1 gene and protein expression, respectively, mean ± SEM. **(D)** Representative double immunolabeling images taken from 17 gw cultures for NR1 (green) with either progenitor, neuronal or astroglial markers (red). After 3 DIV in PM, NR1 was expressed on BLBP+ RGCs, Tbr2+ intermediate progenitors, Nkx2.1^+^ interneuron progenitors, and on GFAP^+^ astroglia. After additional 7 DIV in DM, NR1 was expressed on neurons labeled with βIII tubulin, Tbr1, GABA, and on astrocytes labeled with GFAP. Nuclear stain: BB.

Using the enriched dorsal RGCs cultures (see Materials and Methods; Figure 1), we explored whether isolated RGCs express NMDARs, particularly the obligatory subunit NR1, since KYNA has a specific affinity toward the glycine site of the NR1 subunit (Zhuravlev et al., 2007). Indeed, cultured human RGCs express the NR1 subunit gene (Figures 2A,B) and protein (Figure 2C) after 3 DIV of proliferation and 7 DIV of differentiation in all fetal brains studied (*n* = 4, 16–19 gw). Further, to determine which progenitor subtypes express the NR1 subunit after 3 DIV we used double immunolabeling with antibodies against NR1 and the markers of cortical progenitors (Figure 2D). We demonstrated that RGCs labeled with BLBP^+^, intermediate progenitors labeled with Tbr2^+^, and a subpopulation of interneuron progenitors labeled with Nkx2.1^+^, all express the NR1 subunit. After 7 DIV of differentiation, the newly generated neurons labeled with βIII tubulin, and also with Tbr1 or GABA, markers of glutamatergic and cortical interneurons, respectively, express NR1 (Figure 2D). In humans, GFAP is a marker of both RGCs progenitors and differentiated astrocytes, and both cell types express NR1 subunit. Notably, we observed two types of immunostaining: diffused staining in progenitor cells, possibly due the presence of NR1 in the Golgi network undergoing transport, and punctate staining in neurons, which could reflect membrane localization of NR1 in these cells.

Thus, based on these co-labeling experiments, we conclude that at mid-gestation, both cortical progenitors, two neuronal subtypes, glutamatergic and interneurons, and astroglia cells express the NR1 subunit of NMDARs.

### KYNA affects cell survival and proliferation via blockade of NMDARs

**Figure 3:**
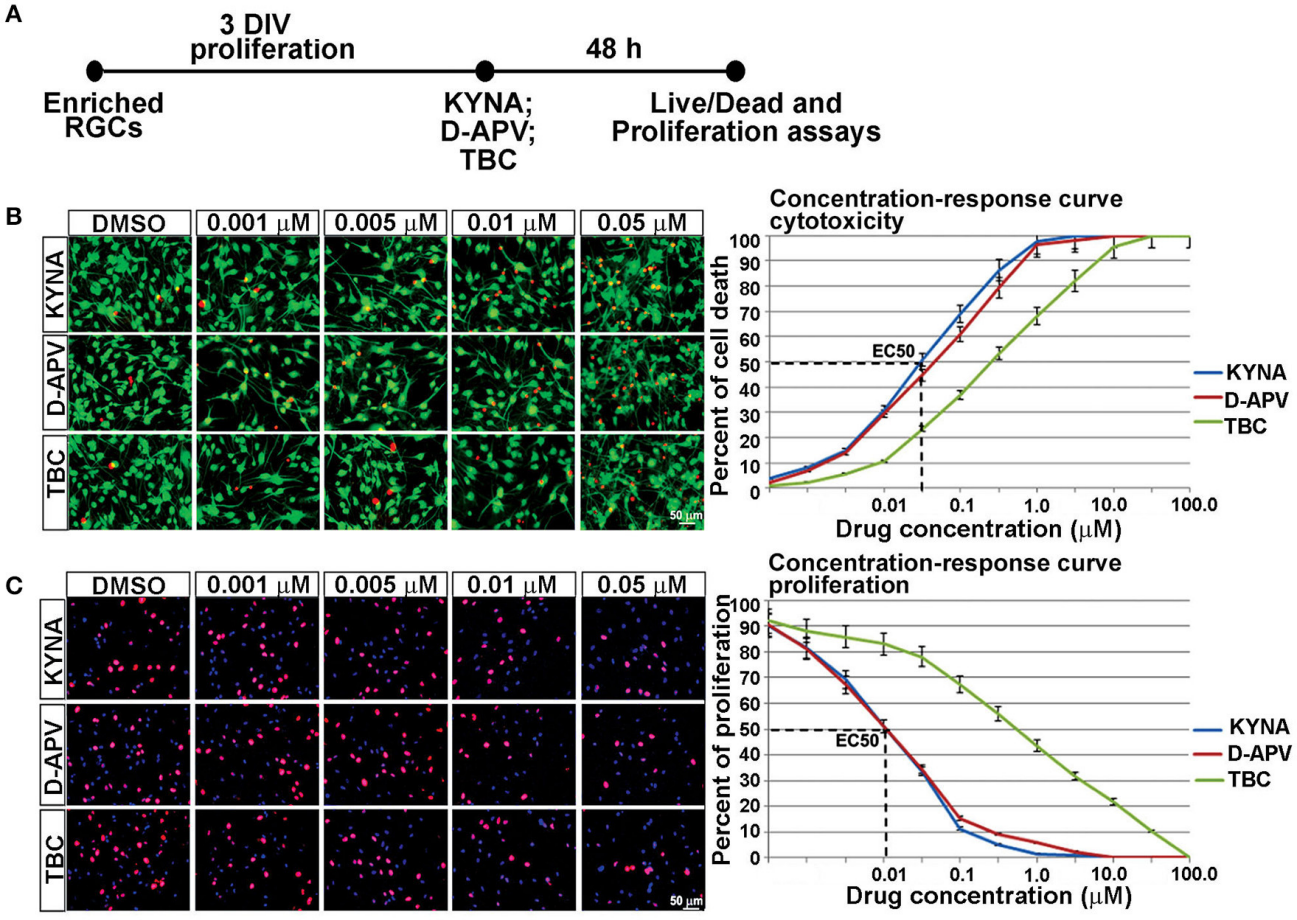
**Concentration-response curve for *in vitro* KYNA treatment. (A)** Timeline of the experiment. **(B)** The live/dead assay: live (green) and dead cells (red) for control (DMSO) and drug treatments. **(C)** Ki67^+^ proliferating cells (red); nuclear stain BB (blue); *n* = 4; *p* < 0.05 between DMSO and each treatment. Mean ± SEM; DMSO, Dimethyl sulfoxide; TBC, Tubocurarine chloride; D-APV, D-amino phosphonovalerate.

We further examined the effects of KYNA on proliferation and survival of human RGCs. KYNA binds to the NR1 subunit of NMDARs as well as to the nAChRs. To determine if the effect of KYNA on cell cycle and cell death was mediated through NMDARs or nAChRs, we used receptor specific antagonists: D-APV specific to NMDARs and TBC for nAChRs (Figure 3A).

Response curves were first plotted 48 h after RGC cultures underwent treatments with eight different concentrations of KYNA, D-APV, and TBC. The effect of KYNA on RGCs proliferation and on cell survival was measured, and the effective concentration at which 50% of the cell population was affected (EC50) was determined in both assays. Considering that the concentration 0.05 μM effectively induced 50% cell death and concentration 0.01 μM effectively inhibited 50% of cell proliferation (Figures 3B,C), we used the following treatment concentrations for further experiments: 0.001, 0.005, and 0.01 μM of KYNA for 48 h. Furthermore, KYNA concentration-response curves in both assays were similar to that of D-APV while TBC curves were shifted to the right, suggesting that in human RGC cultures KYNA exerts most of its effects on cell survival and proliferation in a manner very similar to the NMDAR-specific antagonist D-APV.

### KYNA alters RGC specification

**Figure 4:**
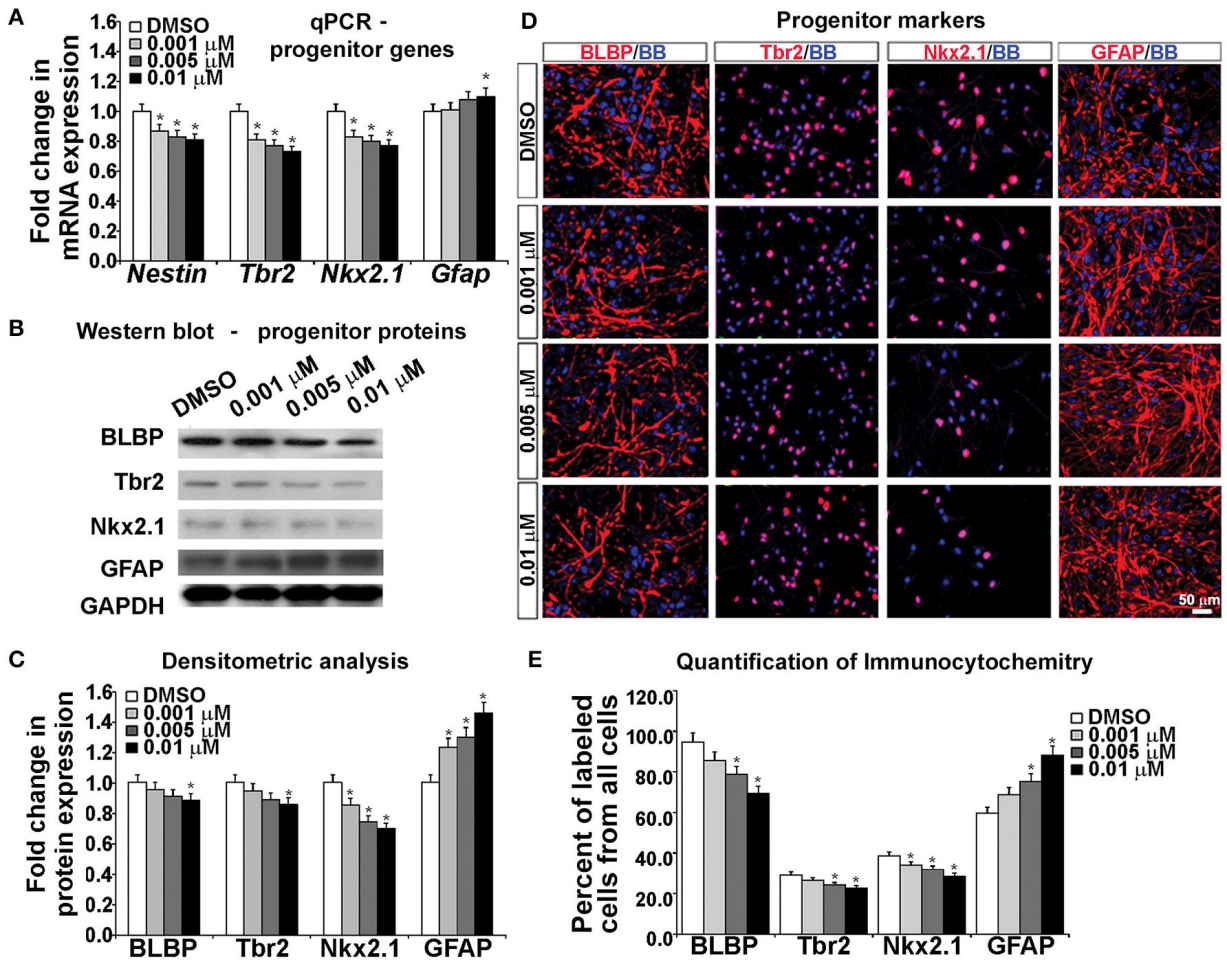
**Blocking NMDAR affects cell fate determination *in vitro*. (A)** KYNA treatment decreased progenitors but increased astroglia specific gene expression in a concentration-dependent manner, **(B)** Western blot from 17 gw KYNA treated cell lysates, and **(C)** the densitometric analysis showed a decrease in progenitor, but increase in astroglia protein expression in a concentration-dependent manner. **(D)** Representative images at 17 gw for immunolabeling with specific progenitor and astroglia markers (red), and nuclear stain: BB (blue). **(E)** The immunolabeling quantification showed a decrease in percentage of progenitor subtypes, but an increase in astroglia population (*n* = 4; ^*^ represents *p* < 0.05). Scale bar 50 μm.

To examine the effects of KYNA on the specification of human cortical progenitors, we followed the progeny of RGCs after 3 DIV of proliferation. Real-time PCR revealed that after KYNA treatment expression of genes specific to progenitor subtypes is decreased in a concentration-dependent manner, while expression of the astroglial gene, *Gfap* is increased with the highest KYNA concentration of 0.01 μM (*p* < 0.05; Figure 4A).

Protein expression, examined in whole cell lysates using Western blotting, revealed that treatment with KYNA significantly reduced expression levels of proteins specific for cortical progenitors. While only the highest concentration of KYNA (0.01 μM) had significant (*p* < 0.05) effects on RGC protein BLBP and intermediate progenitor protein Tbr2, all three concentrations of KYNA reduced expression of protein Nkx2.1 present in subpopulation of cortical interneuron progenitors (*p* < 0.05). In accordance with gene expression levels, astroglial protein GFAP showed an increase after KYNA treatment in a concentration-dependent manner (*p* < 0.05; Figures 4B,C).

Looking at the effects of KYNA on the cell types using immunocytochemistry, we found that the percentage of BLBP^+^ RGCs and Tbr2^+^ intermediate progenitors from all cells in the culture were significantly reduced with the two highest concentrations (0.005 and 0.01 μM), while all three concentrations of KYNA significantly decreased the percentage of Nkx2.1^+^ interneuron progenitors (*p* < 0.05). These results were in line with those observed from the qPCR and Western blotting experiments. Similar to data on gene and protein expression, we found a significant increase in the GFAP^+^ astroglial population in KYNA treated cultures (*p* < 0.05; Figures 4D,E).

These results indicate that KYNA significantly altered the specification of the cortical radial glia progenitor cells by reducing the number of progenitor subtypes (Tbr2, Nkx2.1) while increasing the number of GFAP^+^ astroglial cells, the first type of glia to appear once neurogenesis is completed *in vivo*. This alteration in the progenitor specification timeline is likely to alter the downstream process of neurogenesis and gliogenesis.

### KYNA modifies the process of neuronal differentiation

Since KYNA negatively affected RGCs and altered their specification process, we hypothesized that the process of progenitor cells differentiating into cortical neurons is also affected. In order to test our hypothesis, we added differentiation medium (DM) to our progenitor cell cultures for 7 DIV and then assessed their differentiation into either neurons or astrocytes.

**Figure 5:**
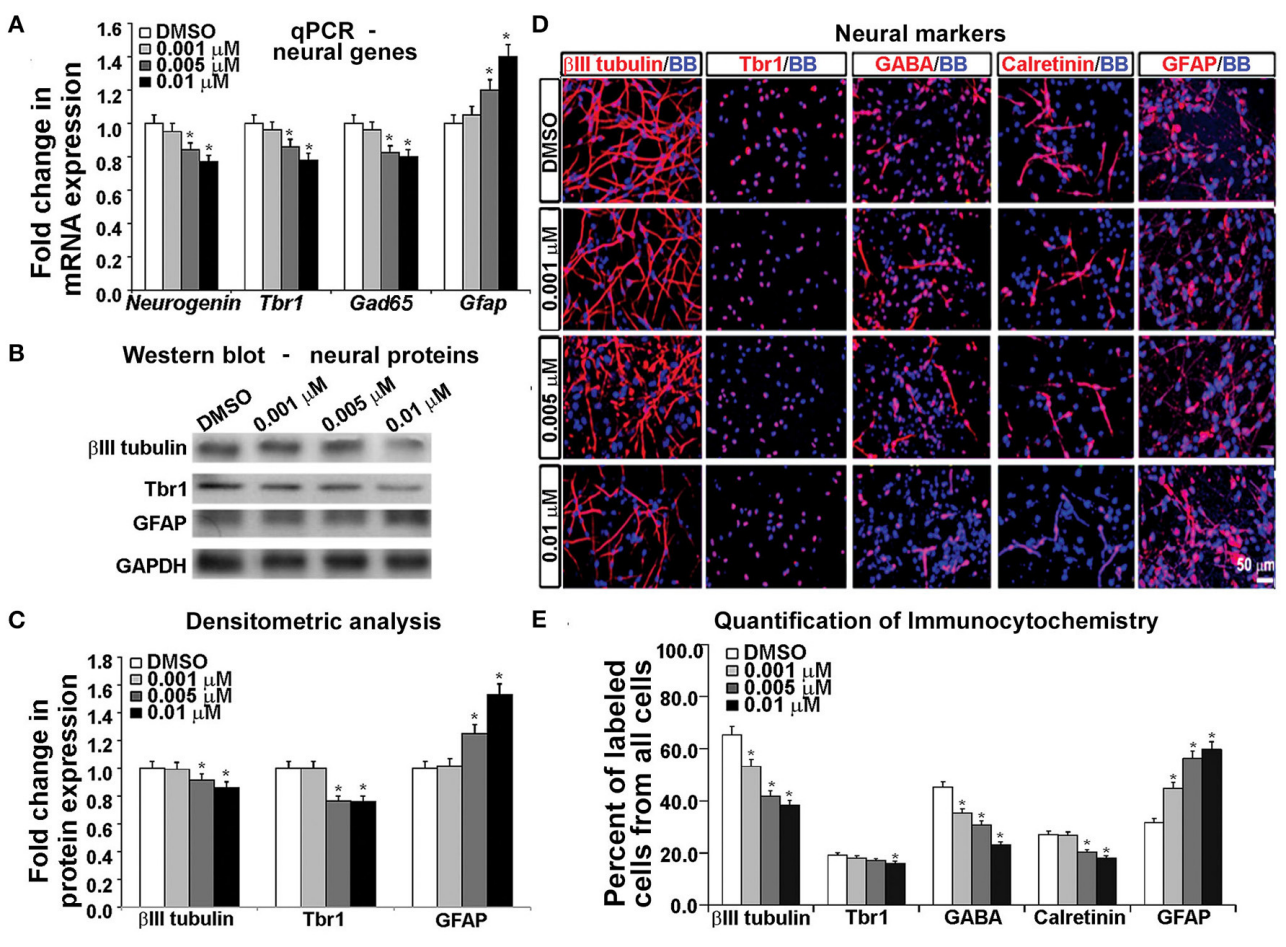
**Differential effect of KYNA treatment on neural cell types. (A)** q-PCR showed that KYNA treatment decreased neuronal specific (*Neurogenin, Tbr1, Gad65*), but increased astrocytic gene (*Gfap*) expression **(B)** Western blot from 17 gw KYNA treated cell lysates, and **(C)** Densitometric analysis showed a decrease in expression for neuron specific proteins, but an increase for astrocytic protein **(D)** Immunostaining at 17 gw for neuron and astrocytic markers (red); nuclei labeled with BB (blue). **(E)** Quantification of the immunolabeled cell types showed a decrease in neuronal cell populations, but an increase in the astrocyte cell population (*n* = 4; ^*^ represents *p* < 0.05).

Gene expression analysis using real time PCR showed that KYNA decreased expression of neuronal genes *Neurogenin 1/2, Tbr1*, and *Gad65* in a concentration-dependent manner, but increased expression of astrocytic gene *Gfap* (*p* < 0.05; Figure 5A). This finding was further supported by the decrease in neuronal proteins βIII tubulin and Tbr1, but an increase in astrocytic protein GFAP after KYNA treatment (*p* < 0.05; Figures 5B,C). Although we demonstrated decrease in mRNA levels in progenitors (Figure 4A) as well as in neurons and astrocytes (Figure 5A), we need to take into account that a pharmacological treatment affects the viability of the cells which limits the interpretation of these results.

To analyze the effect on specific cell-types, we performed immunocytochemistry using antibodies specific to neuronal proteins. We observed a significant decrease in the number of cells labeled with neuronal markers (β-III-tubulin, Tbr1, GABA, Calretinin) and this decrease was KYNA concentration-dependent. Moreover, the decrease of GABAergic neurons was 50% and glutamatergic (Tbr1) neurons 17% comparing to controls, suggesting a greater effect of KYNA on interneuron vs. glutamatergic neurogenesis. Once again, we found an increase in the number of GFAP^+^ astrocytes, indicating a drive toward astrocyte formation (*p* < 0.05; Figures 5D,E). We have not, however, observed any differences in oligodendrocyte progenitors (O4^+^ cells) or mature oligodendrocytes (MBP^+^ cells) in our KYNA treated cultures (not shown).

Combined results obtained with a battery of methods demonstrated that KYNA influenced the RGC specification and differentiation process by increasing the astroglial cell population at the expense of neuronal cells. Developmentally, this shift in gliogenesis occurring before neurogenesis could be detrimental to cortical circuitry formation and function.

### KYNA treatment increases the population of reactive astrocytes

**Figure 6:**
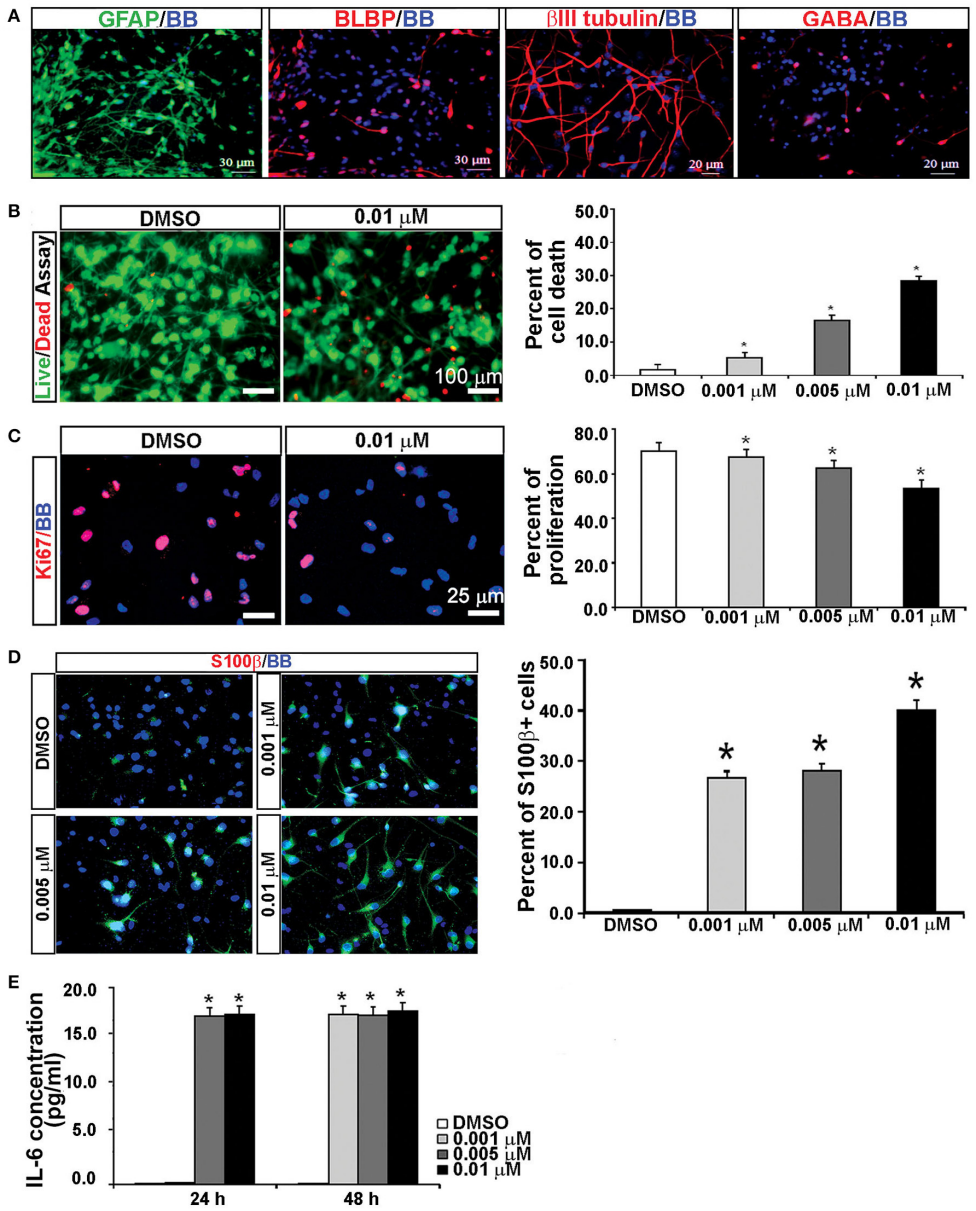
**KYNA treatment activated astrocytes and induced IL-6 secretion in mixed cell cultures. (A)** Mixed cell cultures (18 gw) labeled with RGCs, astrocytes and neuronal markers. **(B)** The live/dead and **(C)** proliferation assays showed that KYNA treatment decreased cell viability and proliferation in a concentration-dependent manner. **(D)** Immunolabeling for S100b^+^ (red). The percentage of S100β^+^ reactive astrocytes increased after 48 h KYNA treatment. **(E)** KYNA treated cultures at 24 and 48 h time points exhibited increase in IL-6 secretion as compared to DMSO control. Nuclear stain: BB; *n* = 4; ^*^ represents *p* < 0.05. Mean ± SEM.

It has been previously reported that KYNA triggers activation of astrocytes and generates an inflammatory response that involves secretion of pro-inflammatory cytokines (Guillemin et al., 2001; Meyer et al., 2011). To test this possibility, we used mixed cell cultures that contain both neurons and glia cells, and have a higher number of astroglial cells (GFAP^+^) than the enriched RGC cultures (Figure 6A; Mo et al., 2007). We treated mixed cortical cultures with the same concentrations of KYNA as used for the enriched RGC cultures, i.e., 0.001, 0.005, and 0.01 μM, for 24 and 48 h and performed cell survival and proliferation assays as previously described. The highest level of KYNA used, 0.01 μM, induced 30% more cell death compared to control (Figure 6B; *p* < 0.05), and decreased cell proliferation by 21.4% compared to control (Figure 6C; *p* < 0.01). We immunolabeled KYNA treated mixed cell cultures with an antibody to S100β, a cytoplasmic marker for reactive astrocytes.

Quantification of the number of S100β^+^ cells immediately after the 48 h KYNA treatment revealed that treatment with KYNA induced a significant increase in S100β^+^ cells, from 0.5% in control conditions to 40% when treated with 0.01 μM KYNA (*p* < 0.001; Figure 6D). These S100β^+^ astrocytes were large cells with multiple processes. Reactive astrocytes are a well-known source of pro-inflammatory cytokines, one of which is IL-6, a primary cytokine released in almost all inflammatory responses (Guillemin et al., 2001; Islam et al., 2009). Since we observed a strong gliosis reaction in our KYNA-treated mixed cell cultures, we determined the level of secreted cytokine IL-6. We measured concentration of secreted IL-6 in the medium at 24 and 48 h after KYNA treatment, and observed significant increase in levels of IL-6 in KYNA treated cultures, at both time points. These changes were concentration-dependent, as shown in Figure 6E. After 24 h, the concentration of IL-6 was increased in cells treated with all three concentrations of KYNA, however, significance was achieved only for concentrations 0.005 μM, and 0.01 μM KYNA (*p* < 0.05), as compared to DMSO (control). After 48 h, the IL-6 concentration was increased in all treated cell cultures regardless of KYNA concentration (*p* < 0.01). The possible reason why we did not observe differences in IL-6 levels between different concentrations of KYNA could be due to the saturation of essay sensitivity in presence of high concentrations of IL6.

## Discussion

In the present study, we explored the effects of KYNA on human cerebral cortical development in cell cultures derived from mid-gestational human tissue. Previous studies in rodents (Malatesta et al., 2000; Miyata et al., 2001; Noctor et al., 2001, 2004), and humans (Clowry et al., 2010; Hansen et al., 2010; Betizeau et al., 2013; Malik et al., 2013; Radonjić et al., 2014) have shown significant species-specific differences demonstrating critical need for studies of cortical development in human cultures. Three major findings from this study demonstrate that KYNA, acting as an antagonist of NMDARs, significantly alters proliferation, specification and differentiation of cortical cells generated from enriched RGC cultures. First, after KYNA treatment, RGC proliferation and survival decreased resulting in a reduced number of cortical progenitors (Nkx2.1^+^ and Tbr2^+^ cells), neurons (βIII tubulin^+^, Tbr1^+^, CalR^+^), and interneurons (GABA^+^). Second, KYNA treatment effectively pushed the specification of RGCs toward the astroglial lineage, as evidenced by the increase in number of GFAP^+^ cells after 3 DIV of proliferation. Not only did the number of GFAP^+^ astrocytes increase, but in addition astrocytes were activated as demonstrated by morphological changes and S100β labeling. Third, KYNA exposure triggered an inflammatory response as evidenced by increased levels of the pro-inflammatory cytokine IL-6. In sum, these results indicate that altered levels of KYNA during human gestation, may have profound effects on cortical development, and through activation of astrocytes, initiate an inflammatory response resulting in a positive feedback loop.

### KYNA disrupts neurogenesis and reduces the pool of cortical progenitors *in vitro*

KYNA is a product of the kynurenine pathway, the main metabolic route of tryptophan degradation, and is responsible for a broad spectrum of effects, including the endogenous regulation of neuronal excitability and the initiation of immune tolerance. KYNA is the only known endogenous antagonist of the NMDAR *in vivo*, and binds strongly to the NR1 subunit (Kessler et al., 1989; Wang et al., 2006). However, KYNA also inhibits α7nAChRs (Hilmas et al., 2001; Alkondon et al., 2004), and both NMDAR and nAChRs are expressed early during human development (4–5 gw; Kostović et al., 1989; Hellström-Lindahl et al., 1998; Suzuki et al., 2006). Therefore, it was necessary to distinguish which receptor mediates KYNA effects on the early developing brain. In this study, we showed that in RGCs, KYNA acts similar to NMDAR-specific antagonist D-APV, corroborating previous studies that showed KYNA's inability to block nAChR-dependent electrical activity in cultured adult rat cortical and hippocampal cells (Hilmas et al., 2001; Mok et al., 2009; Dobelis et al., 2012).

During embryonic development in rodents and humans, NMDARs are important for proliferation of neural stem cells, their differentiation into neurons and glia, and proper migration of the differentiated cell types to their correct position in the six-layered cerebral cortex (Manent et al., 2005; Suzuki et al., 2006; Toriumi et al., 2012). *In vivo* blockade of NMDARs during rat cortical development induces apoptosis of neuronal and glial cells leading to severe neurodegeneration and ultimately death of the animal (Ikonomidou et al., 1999). *In vitro*, we observed that antagonizing NMDARs with KYNA increases cell death of cortical progenitors and decreases RGC proliferation, showing the importance of NMDARs for both survival and proliferation of RGCs in the human cerebral cortex during the second trimester of gestation.

### KYNA-induced changes in cell fate determination

An important issue is whether KYNA has differential effects on generation of principal neurons and interneurons in the developing cortex. To study this question, we used various transcription factors to label distinct neuronal populations *in vitro*. Transcription factor Nkx2.1 is a marker of a subpopulation of cortical interneuron progenitors (Rakic and Zecevic, 2003; Zecevic et al., 2005; Jakovcevski et al., 2011), and Tbr2 is a marker of glutamatergic progenitors in the developing human cerebral cortex (Bayatti et al., 2008; Yu and Zecevic, 2011; Malik et al., 2013; Pollen et al., 2015). This study confirmed our previous results that human RGCs have a capacity to generate both Nkx2.1^+^ and Tbr2^+^ cells *in vitro* (Yu and Zecevic, 2011) and indicated further commitment of RGCs to both interneuron and glutamatergic lineages. For example, after allowing time for differentiation, a percentage of fetal cortical RGCs start expressing GABA a major interneuron transmitter. On similar grounds, after differentiation cortical RGCs were shown to express Tbr1, a transcription factor downstream of Tbr2, indicating commitment to the glutamatergic lineage (Englund et al., 2005). Most notably, we have shown that a KYNA-induced decrease in progenitors (Nkx2.1 and Tbr2) resulted further in a decrease of GABAergic interneurons and Tbr1^+^ glutamatergic neurons. Notably, the decrease of GABAergic neurons was more pronounced than that of glutamatergic neurons. These data suggest that a KYNA-induced alteration in the cortical progenitor pool disrupts neurogenesis and may affect the proportional balance of cortical cell types and subsequent formation of cortical circuitry.

### KYNA elicits pro-inflammatory response

Mounting evidence suggests a link between maternal immune activation and neuropsychiatric illness (Gilmore and Jarskog, 1997; Buka et al., 2001). The KYNA-mediated NMDAR blockade favored astrocyte generation, as had been shown previously in human fetal astrocyte cultures (Guillemin et al., 2001), and in addition, induced the activation of resting astrocytes in both RGC cultures and mixed cell cultures. The morphology of S100β+ cells changed, and their number increased, implying a pro-inflammatory response. Interestingly, astrocytic hypertrophy has been reported in the prefrontal cortex of patients with bipolar disorder (Rajkowska et al., 2001), indicating that reactive astrocytic changes can be a major component of neuropsychiatric illness.

We also showed increased levels of secreted IL-6, a pro-inflammatory cytokine, in our human culture medium following KYNA treatment. This is in agreement with reports in mice that KYNA causes the release of inflammatory cytokines, especially IL-6 and IFN-γ (Islam et al., 2009). Moreover, *in vitro*, both IFN-γ and IL-6 activate human fetal astrocytes to synthesize large amounts of KYNA (Guillemin et al., 2001; Islam et al., 2009; Meyer et al., 2011; Schwieler et al., 2015). Activation of cytokines is directly relevant to schizophrenia as studies in human subjects found elevated levels of IL-6 in the cerebrospinal fluid of Sch patients in comparison to their age and sex-matched healthy counterparts (Garver et al., 2003; Schwieler et al., 2015). With respect to the potential developmental pathways involved, KYNA has been shown to elicit a pro-inflammatory response that can activate the JAK-STAT pathway in mice (Schwarcz et al., 2001; Erhardt et al., 2012; Holtze et al., 2012). This would result in cytokine-receptor interactions and could interfere with the normal specification of cortical progenitors at mid-gestation.

### Consequences of elevated KYNA levels during fetal neurodevelopment

Metabolites of the kynurenine pathway have important roles in the CNS, and recent data imply a role in brain development as well (reviewed in Notarangelo and Pocivavsek, 2016). Physiological levels of KYNA are higher in the developing brain compared to the immediate post-natal period and adulthood (Beal et al., 1992; Walker et al., 1999; Ceresoli-Borroni and Schwarcz, 2000). It has been suggested that KYNA might protect the fetal brain from over-excitation via NMDARs (Badawy, 2014). At the same time, glutamate via activation of NMDARs has an important trophic role in neurodevelopment. Various insults during pregnancy can lead to direct physiological changes in the fetal environment, thereby influencing the normal course of prenatal brain development. For example, the anesthetic ketamine and drugs of abuse such as PCP and ethanol antagonize normal function of fetal cortical NMDARs (Anis et al., 1983; Krystal et al., 1994; Lahti et al., 1995; Lieberman et al., 2012; Xiang et al., 2015). These disruptions may have long-lasting consequences for subsequent brain and behavioral development and might lead to structural and functional brain abnormalities in adult life (Iaccarino et al., 2013).

Even though it is well established that KYNA acts as an antagonist of NMDAR and nAChR, the consequences of pathological levels of KYNA in the developing brain have not been fully explored. Our *in vitro* study shows that KYNA, by antagonizing NMDARs, negatively influences the proliferation of human RGCs and pushes RGC specification toward astrocytes, vs. neurons. In addition, higher KYNA concentrations, while negatively impacting both interneurons and principle neurons, results in larger deficits in GABA^+^ interneurons compared to a Tbr1^+^ subpopulation of projection neurons. The preferential vulnerability of interneurons might be clinically relevant since impaired function of cortical interneurons is reported in neurodevelopmental disorders including Sch and autism spectrum disorder (Akbarian et al., 1995; DeFelipe, 1999; Knable, 1999; Gleeson and Walsh, 2000; Lewis and Levitt, 2002; Levitt, 2003; Baraban and Tallent, 2004; Lewis et al., 2005). In our study, human cortical progenitors were isolated at mid-gestation (16–19 gw), an important period of active neurogenesis when upper cortical layers are formed (Hill and Walsh, 2005). As these layers are crucial for formation of cortico-cortical connections and subsequently for emergence of higher brain functions, interference with NMDARs that are expressed on all cortical cell types (Bagasrawala et al., 2016) at this developmentally sensitive period may lead to a widespread effect on formation of the cortical circuitry. Together, these changes can have a negative impact on cortical functional maturation during adolescence, a stage when cognitive executive functions, such as planning, rational thinking, attention, and emotion-related impulsivity control develop, and a time point at which Sch symptoms often first manifest (Uhlhaas, 2011). The effects of KYNA on human cortical progenitors *in vitro*, reported in this study, are still preliminary. Further studies are, therefore, needed to determine if KYNA exerts its effect on RGCs by a selective or inductive action mechanism.

Certainly, *in vitro* systems have limitations, and further studies are needed to discern the complexity of developmental disruption caused by NMDAR antagonism. However, the *in vitro* system studied here serves as an innovative tool for understanding the possible effects of endogenous molecules, when present at pathologic levels, on human fetal cortical development. This method enables us to identify the specific cellular targets of KYNA but also to determine possible cell signaling molecules involved in human cortical development.

## Author contributions

IB did all experiments, analyzed data and was involved in writing the results. NZ substantially contributed in all aspect of this study. NR substantially contributed in design of the study, analysis and writing the results.

## Funding

This work was supported by NIH grants 2R01NSO41489 and subcontract 5R01DA023999-07(NZ).

### Conflict of interest statement

The authors declare that the research was conducted in the absence of any commercial or financial relationships that could be construed as a potential conflict of interest.
